# Improved prediction of outcome in Parkinson's disease using radiomics analysis of longitudinal DAT SPECT images

**DOI:** 10.1016/j.nicl.2017.08.021

**Published:** 2017-08-26

**Authors:** Arman Rahmim, Peng Huang, Nikolay Shenkov, Sima Fotouhi, Esmaeil Davoodi-Bojd, Lijun Lu, Zoltan Mari, Hamid Soltanian-Zadeh, Vesna Sossi

**Affiliations:** a Department of Radiology, Johns Hopkins University, Baltimore, United States; b Department of Electrical & Computer Engineering, Johns Hopkins University, Baltimore, United States; c Departments of Oncology and Biostatistics, Johns Hopkins University, Baltimore, United States; d Department of Physics & Astronomy, University of British Columbia, Vancouver, Canada; e Departments of Radiology and Research Administration, Henry Ford Health System, Detroit, MI, United States; f School of Biomedical Engineering, Southern Medical University, Guangzhou, China; g Department of Neurology and Neurosurgery, Johns Hopkins University, Baltimore, MD, United States; h CIPCE, School of Electrical & Computer Engineering, University of Tehran, Tehran, Iran

**Keywords:** DAT SPECT, Longitudinal, Radiomics, Textural features, Outcome prediction, Parkinson's disease

## Abstract

No disease modifying therapies for Parkinson's disease (PD) have been found effective to date. To properly power clinical trials for discovery of such therapies, the ability to predict outcome in PD is critical, and there is a significant need for discovery of prognostic biomarkers of PD. Dopamine transporter (DAT) SPECT imaging is widely used for diagnostic purposes in PD. In the present work, we aimed to evaluate whether longitudinal DAT SPECT imaging can significantly improve prediction of outcome in PD patients. In particular, we investigated whether radiomics analysis of DAT SPECT images, in addition to use of conventional non-imaging and imaging measures, could be used to predict motor severity at year 4 in PD subjects. We selected 64 PD subjects (38 male, 26 female; age at baseline (year 0): 61.9 ± 7.3, range [46,78]) from the Parkinson's Progressive Marker Initiative (PPMI) database. Inclusion criteria included (i) having had at least 2 SPECT scans at years 0 and 1 acquired on a similar scanner, (ii) having undergone a high-resolution 3 T MRI scan, and (iii) having motor assessment (MDS-UPDRS-III) available in year 4 used as outcome measure. Image analysis included automatic region-of-interest (ROI) extraction on MRI images, registration of SPECT images onto the corresponding MRI images, and extraction of radiomic features. Non-imaging predictors included demographics, disease duration as well as motor and non-motor clinical measures in years 0 and 1. The image predictors included 92 radiomic features extracted from the caudate, putamen, and ventral striatum of DAT SPECT images at years 0 and 1 to quantify heterogeneity and texture in uptake. Random forest (RF) analysis with 5000 trees was used to combine both non-imaging and imaging variables to predict motor outcome (UPDRS-III: 27.3 ± 14.7, range [3,77]). The RF prediction was evaluated using leave-one-out cross-validation. Our results demonstrated that addition of radiomic features to conventional measures significantly improved (*p* < 0.001) prediction of outcome, reducing the absolute error of predicting MDS-UPDRS-III from 9.00 ± 0.88 to 4.12 ± 0.43. This shows that radiomics analysis of DAT SPECT images has a significant potential towards development of effective prognostic biomarkers in PD.

## Highlights

• Aim to enable image-assisted prediction of outcome in Parkinson's disease.
• Radiomics analysis of clinical dopamine transporter (DAT) SPECT images (DaTscans).
• Significant improvement in prediction of motor outcome.

## 1 Introduction

Parkinson's disease (PD) is a progressive, degenerative movement disorder, characterized by neuronal loss in the substantia nigra with the loss of dopaminergic terminals in the basal ganglia (Brooks et al., 1990, Garnett et al., 1987, Stoessl et al., 2011). Given the absence of proven disease modifying therapies for PD, there is a critical need to establish biomarkers of disease progression (Marek et al., 2008); e.g. an aim of the Parkinson's Progressive Marker Initiative (PPMI) (Parkinson Progression Marker Initiative, 2011). Furthermore, there is significant interest in prognostication of disease outcome, to properly adapt and power clinical trial studies, as applied to appropriate patients. Stratification of PD based on expected prognosis would allow better designs of disease modifying trials, with greater power to ascertain efficacy.

Imaging of the dopaminergic system with ^123^I-ioflupane-dopamine transporter (DAT) SPECT is now widely used (Catafau and Tolosa, 2004, Grachev et al., 2012, Kupsch et al., 2012). DAT SPECT images are commonly assessed visually. However, there are increasing efforts to quantify uptake in regions-of-interest (ROIs) in order to provide more objective measures of disease severity (Badiavas et al., 2011, Koch et al., 2005). Quantitative imaging biomarkers also have the potential for earlier detection of disease. A significant way in which DAT SPECT imaging has been helpful is to identify a subgroup of PD patients who are symptomatic without evidence of dopamine deficit (SWEDDs). These patients have a significantly better prognosis. What remains of critical importance, is to discover further subsets in the PD population of different outcomes, to enable significantly improved targeted clinical trials for the assessment of novel therapies for PD.

Advanced radiographic metrics that quantify heterogeneity in shape and uptake have been explored, primarily in the field of oncology, in order to improve diagnosis as well as prediction of treatment response and survival outcome in different cancers (Eary et al., 2008, El Naqa et al., 2009, Hatt et al., 2015, Rahmim et al., 2016b, Soufi et al., 2017, Tixier et al., 2014, Tixier et al., 2011, van Velden et al., 2011, Vriens et al., 2012). Overall, the field of *radiomics* aims to extract a large number of quantitative features from radiological images, aiming to uncover correlates of disease characteristics that are ordinarily not visually observed or quantitatively measured (Aerts et al., 2014, Asselin et al., 2012, Chicklore et al., 2013, Kumar et al., 2012, Lambin et al., 2012). In a previous work, we assessed whether radiomics analysis improved correlations with clinical assessments (Rahmim et al., 2016a). The present work investigates whether radiomics analysis significantly adds to the ability of non-imaging and conventional measures to predict outcome. This is plausible since pathophysiologic studies have demonstrated very heterogeneous patterns of dopamine loss in the basal ganglia (Kish et al., 1988). As such, we hypothesize that radiomics analysis has the potential to significantly improve prediction of outcome in Parkinson's disease patients.

## 2 Methods and materials

### 2.1 Longitudinal patient data

Longitudinal data were extracted from the PPMI database (www.ppmi-info.org/data) (Parkinson Progression Marker Initiative, 2011). The movement disorder society – unified Parkinson's disease rating scale (MDS-UPDRS) – part III (motor) in year 4 was used as outcome (referred to as UPDRS-III from here on). Predictors included demographics (age, sex), as well as baseline (year 0) and year 1 DAT SPECT images and clinical measures. Clinical measures included: disease duration (DD) taken with respect to (a) time of diagnosis (DD-diag.) as well as (b) time of appearance of symptoms (DD-sympt.). This also included (c) the motor measure, UPDRS-III, and (d) the non-motor cognitive measure, Montreal Cognitive Assessment (MoCA), for both baseline (year 0) and year 1. For consistency, we only included patients with SPECT data acquired on similar kinds of scanner (Siemens, 2-headed ECAM or Symbia systems), and subjects who underwent high-resolution 3 T MRI. These selection criteria resulted in 64 PD subjects (38 male, 26 female; age at year 0: 61.9 ± 7.3, range [46,78]), with widely distributed year 4 outcome UPDRS-III: 27.3 ± 14.7; range [3,77].

Imaging was performed 4 ± 0.5 h following injection of DAT SPECT (^123^I-Ioflupane; 111–185 MBq). Thyroid update was blocked via pre-treatment of subjects with saturated iodine solution (10 drops in water) or perchlorate (1000 mg) prior to injection. Data acquisition consisted of 128 × 128 raw SPECT projection data acquired every 3 degrees, 120 projections, 20% symmetric photopeak windows centered on 159 keV and 122 keV, and a total scan duration of ~ 30–45 min. A HERMES system (Hermes Medical Solutions, Stockholm, Sweden) was used to perform iterative OSEM reconstruction on the input raw SPECT projection data, for all studies to ensure consistency.

Subsequently, PMOD (PMOD Technologies, Zurich, Switzerland) was used for attenuation correction. Ellipses where drawn on the images and Chang 0 attenuation correction was applied invoking a site-specific mu as empirically derived from phantom data (as acquired in site initiation for the trial). Following this, standard 3D Gaussian post-smoothing (6.0 mm FWHM) was applied.

### 2.2 Image processing and quantification

Image processing consisted of segmentation of MRI images and registration of SPECT images onto MRI images, followed by quantitative feature extraction from SPECT images, as follows:

1) *Segmentation*: We focused on six ROIs, namely the caudate, putamen and ventral striatum (VS) (both right and left) for analysis. The occipital cortex was also segmented, which is commonly used as a reference region for normalization of counts (Badiavas et al., 2011, Djang et al., 2012, Koch et al., 2005). Segmentation consisted of: 1) affine registration of the T1-weighted MRI of the subject to the MNI305 atlas (Evans et al., 1993); 2) initial volumetric labeling of the registered MRI of the subject using the labels of the atlas; 3) correction of image inhomogeneity, i.e., variation of the image intensity due to the B1 bias field, using the results of the previous stage; 4) nonlinear volumetric alignment of the subject's affine-registered and inhomogeneity-corrected MRI to the MNI305 atlas; and 5) propagation of the atlas labels to the MRI generated in the previous step and then, mapping of the results back to the original MRI of the subject. The classification of each point in the space to a given label was achieved by finding the segmentation that maximized the probability of input (image) given the prior probabilities from the training set (MNI305 atlas). Details of this framework are presented by Fischl et al., and an implementation of the methods is available in FreeSurfer (Fischl et al., 2002, Fischl et al., 2004a, Fischl et al., 2004b).
2) *Registration*: To register SPECT images of each subject to the MRI of the subject, we employed a rigid, information-theory-based co-registration approach (Penny et al., 2011), implemented in two steps. The algorithm uses the normalized mutual information as the objective function along with a Gaussian smoothing kernel with a width of 7 mm. It also uses two separation levels, the average distance between sampled points of 4 and 2 mm, for the coarse to fine registration of the images, as elaborated by Collignon et al. (1995). Since DAT SPECT images have lower spatial resolution (typically 10 mm) compared to MRI (typically 1 mm), and depict centralized hyper intense striatal uptake (with minimal uptake elsewhere), standard co-registration commonly performs poorly. To tackle this, in the next stage, we performed linear intensity normalization of the T1-w images so that the average intensity of white matter equals 100. Then, we set the intensity value of the caudate and VS to 4000 and putamen to 1000. Finally, we employed the rigid co-registration algorithm on the co-registered DAT SPECT images from the previous stage and the manipulated T1-w images. In this way, the hyper intensity regions in the SPECT image are forced to better align to the regions around the favorable structures. In the end, we overlaid the structures segmented in MRI on the co-registered SPECT images. Furthermore, the more (m) vs. less (l) affected sides of the structures were determined with reference to the SPECT uptake at the putamen.
3) *Feature extraction*: A total of 92 imaging features were extracted, including 13 first-order intensity features, 22 morphological features, and 57 second- and higher-order textural features, describing the intensity and spatial distribution of radiotracer uptake. The definitions of these features are elaborated in the supplement. Of the 92 features, 75 of them incorporated DAT SPECT uptake information, while 17 of the morphological features were purely based on the MRI-based ROIs (see supplement). This latter is justified given findings of structural brain atrophy in PD patients (Brenneis et al., 2003, Zeighami et al., 2015). Of the 92 features, 4 features were conventional measures of SUVmax, SUVmean, SUVpeak and ROI-volume, though these were extracted for each of the 6 ROIs and on both years of imaging (year 0 and 1).

### 2.3 Data analysis

Multivariate analysis was performed in three groups, involving: (1) use of only non-imaging features (i.e. demographics and clinical measures), (2) additional use of conventional imaging features, and (3) additional use of radiomic features. For each group of predictors, a single regression tree was fitted to explore interactions among variables, followed by random forest analysis (R package *randomForest*) to identify the top predictors. Random forest analysis is an ensemble non-parametric machine learning method. It can handle complicated interactions among large number of variables and their non-linear effects efficiently. In fact, it has shown favorable performance in comparison to other machine learning methods in the context of radiomics analysis (Parmar et al., 2015). An observation is that the prediction model cannot be visualized using a single straightforward formula because the prediction is done through a collection of trees, and each tree has its own formula, which is also the case with artificial neural networks (ANNs).

Year 4 UPDRS-III was used as the response variable in all these analyses. Features with zero variance were removed before the analysis. For each group of predictors, 5000 bootstrap samples were randomly drawn from all patients. Each of these bootstrap samples included about two-third of the total number of patients, and the remaining one-third patients were called out-of-bag (OOB) for that bootstrap sample. A decision tree was grown from each bootstrap sample and internally validated using the corresponding OOB sample. This procedure was repeated for all bootstrap samples. The minimum node size was set to 5, and at each node, 1/3 of candidate features were randomly selected to determine how to split the node. To rank the importance of a predictor, all values of this predictor were permuted among all individuals in the OOB, and we put both permuted and non-permuted data down all trees to obtain their predictions. The average absolute difference in prediction between permuted and non-permuted OOB data was then used to measure the variable importance score and to select top predictors. Variables with larger difference scores were considered more important.

Prediction accuracy of random forest analysis was assessed using leave-one-out-cross-validation (LOOCV). Each time, one patient was excluded from the random forest analysis. The developed random forest algorithm was then applied to this excluded patient to obtain a prediction of UPDRS-III score. We then calculated the absolute difference between the predicted and observed UPDRS-III scores for the patient. The process was repeated for all patients. The average difference Δ from all patients was used to compare predictions using different groups of predictors. In particular, we investigated added value of radiomics features by comparing Δ from group 2 and group 3. For longitudinal analysis, combined features from year 0, year 1, as well as their differences were utilized. To assess performance when only baseline information was included, the abovementioned analysis was repeated when data from only year 0 were used to predict outcome in year 4.

## 3 Results

**Fig. 1:**
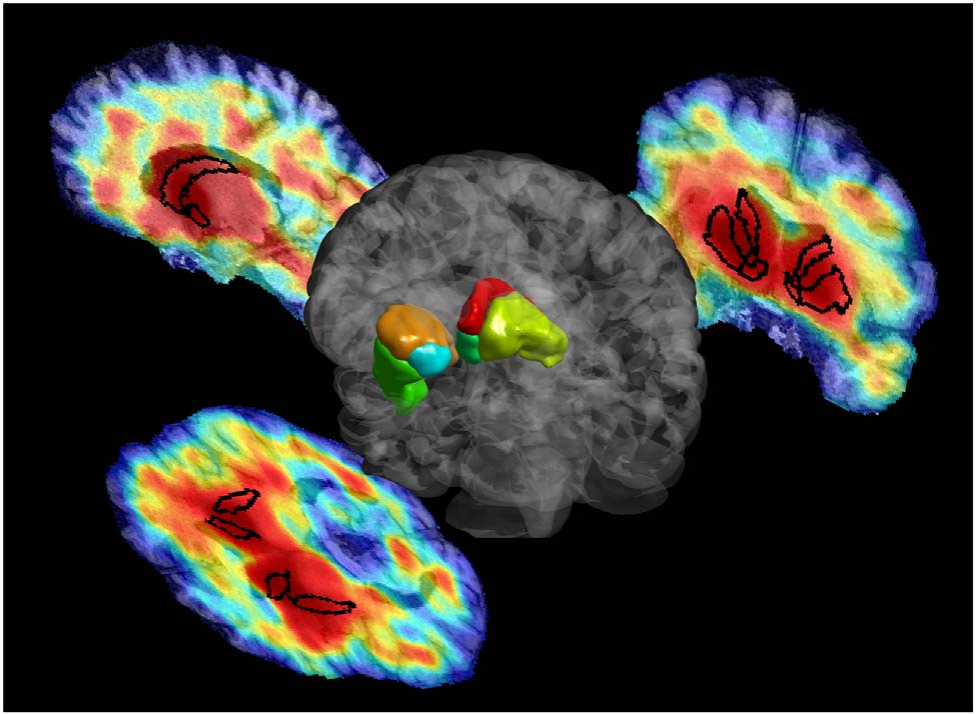
3D volume rendering of six segmentations (caudate, putamen and VS; both right and left) for a typical study, as well as transaxial, coronal and sagittal slices through the DAT SPECT image with superimposed segmentations.

Fig. 1 depicts 3D segmentation as performed on a typical study, as well as transaxial, coronal and sagittal slices of the DAT SPECT image overlaid with the segmentations.

**Fig. 2:**
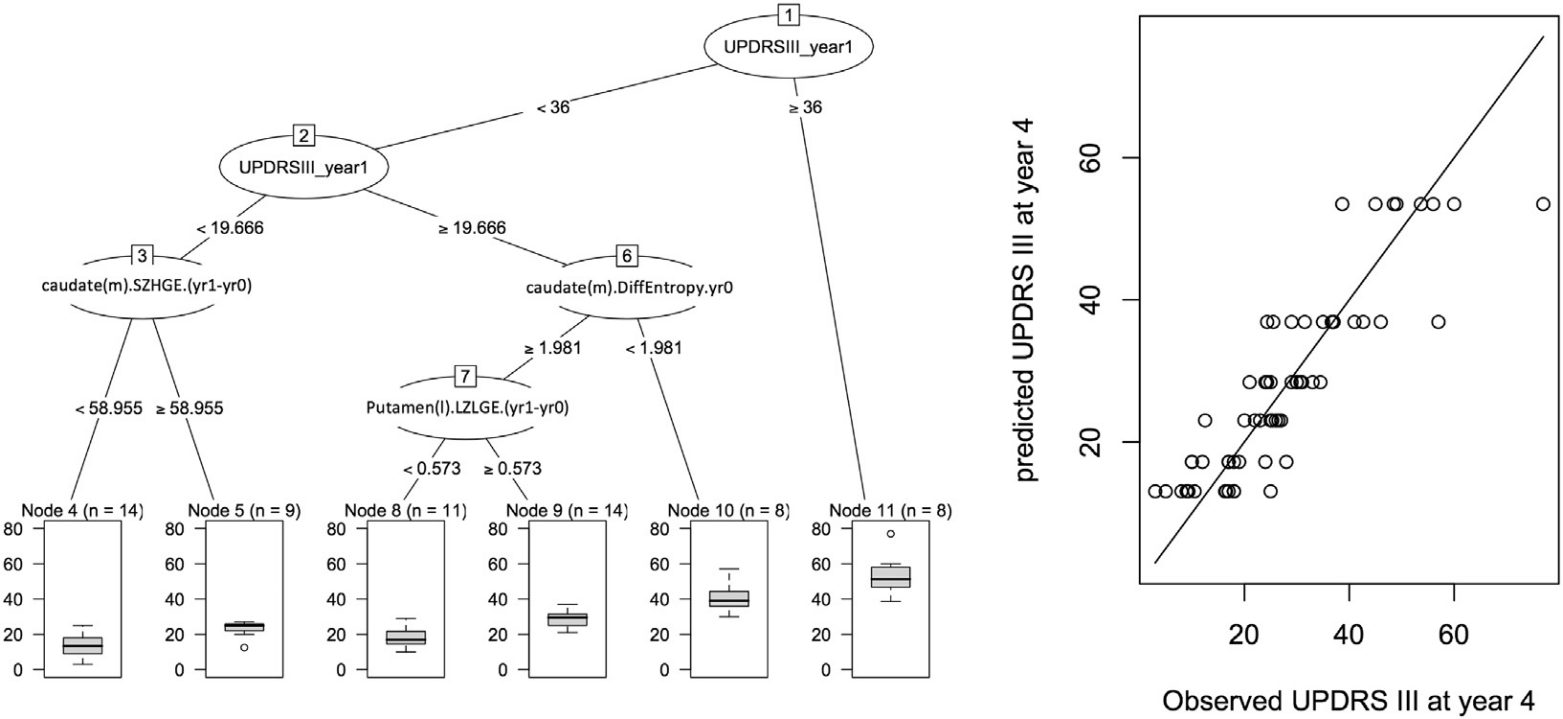
(left) A decision tree, with six leaves, for prediction of UPDRS-III motor outcome. (right) The performance of the tree on the data is shown, which is sub-optimal, given that only one of six outcomes can be arrived at, at the leaves. Use of random forest of decision trees aims to improve this performance. Radiomic features, such as difference Entropy, SZHGE and LZLGE as seen above, are elaborated in the supplement. (m) and (l) refer to the more and less affected sides, respectively (e.g. caudate(m) is the more affected caudate).

Fig. 2 shows a single decision tree (left) and its performance (right). Patients with baseline UPDRS-III greater than or equal to 36 had the highest median year 4 UPDRS-III score. For patients with baseline UPDRS-III < 36, there were interactions among different radiomic features. However, it is seen that using a single decision tree results in a limited number of outcomes, which is why a random forest of decision trees is utilized to provide improved performance (next).

**Fig. 3:**
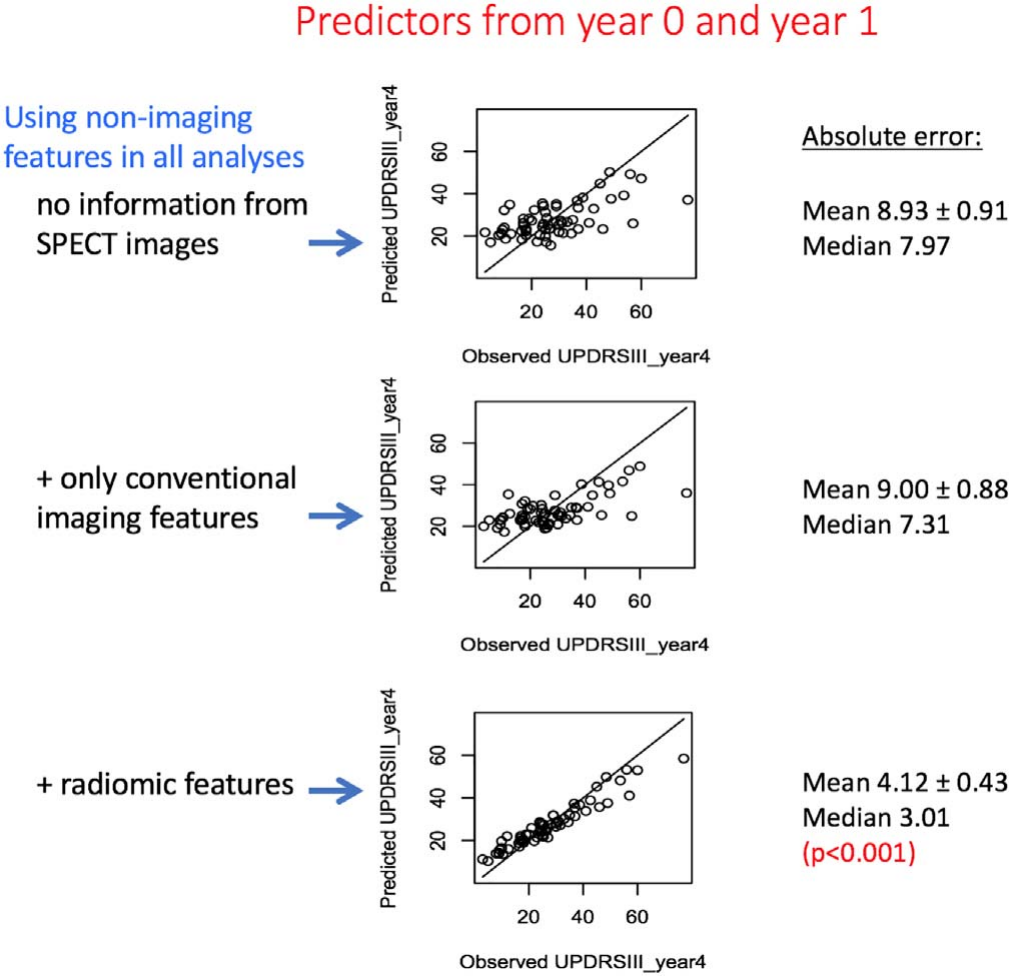
Plots of outcome prediction, when using (top) only demographics and clinical measures, (middle) addition of conventional features as extracted from DAT SPECT images, and (top) addition of radiomics features as extracted from the images. Data from years 0 and 1 are both utilized to predict motor performance in year 4.

Fig. 3 depicts our main findings. Non-imaging features (specifically, demographics and clinical motor and non-motor measures) are used in all predictions. When only these measures are used, absolute error in prediction of outcome is 8.93 ± 0.91. It is seen that addition of only conventional imaging features does not improve prediction performance (9.00 ± 0.88). However, addition of radiomic features results in an absolute error in outcome prediction of 4.12 ± 0.43. Given the year 4 UPDRS-III distribution (27.3 ± 14.7), this represents a significant (*p*-value < 0.001) average improvement of 18% in prediction of outcome, and can be readily observed visually.

**Fig. 4:**
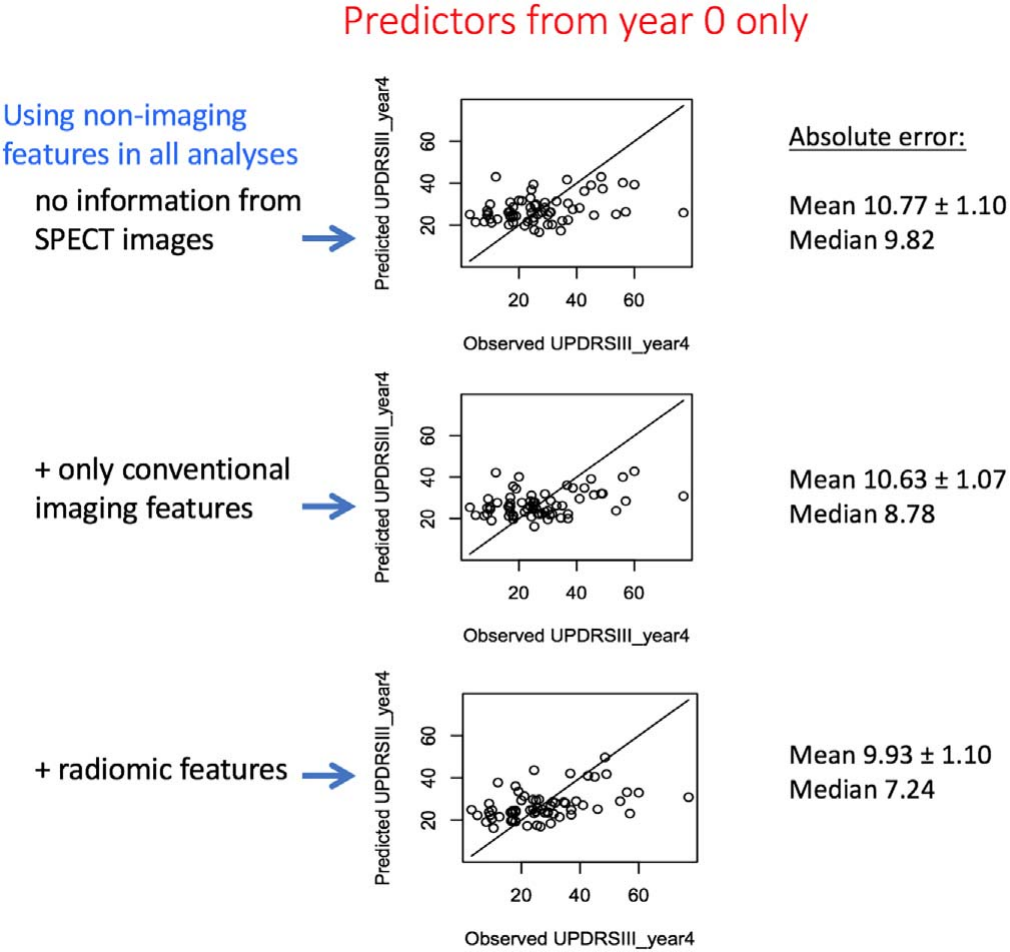
Plots of outcome prediction, when using (top) only demographics and clinical measures, (middle) addition of conventional features as extracted from DAT SPECT images, and (top) addition of radiomics features as extracted from the images. Data from only year 0 (baseline) are utilized to predict motor performance in year 4.

Fig. 4 depicts the findings when data from only year 0 (baseline) are used to predict outcome. It is generally seen that predictions are poorer. What is more, in comparison to use of non-imaging features (10.77 ± 1.10), outcome prediction it not improved when adding conventional features (10.63 ± 1.07) or even by further addition of radiomic feature (9.93 ± 1.10). Overall, it is seen that longitudinal images (acquired at year 0 and year 1) are required to enable significantly improved prediction of motor outcome in year 4.

**Fig. 5:**
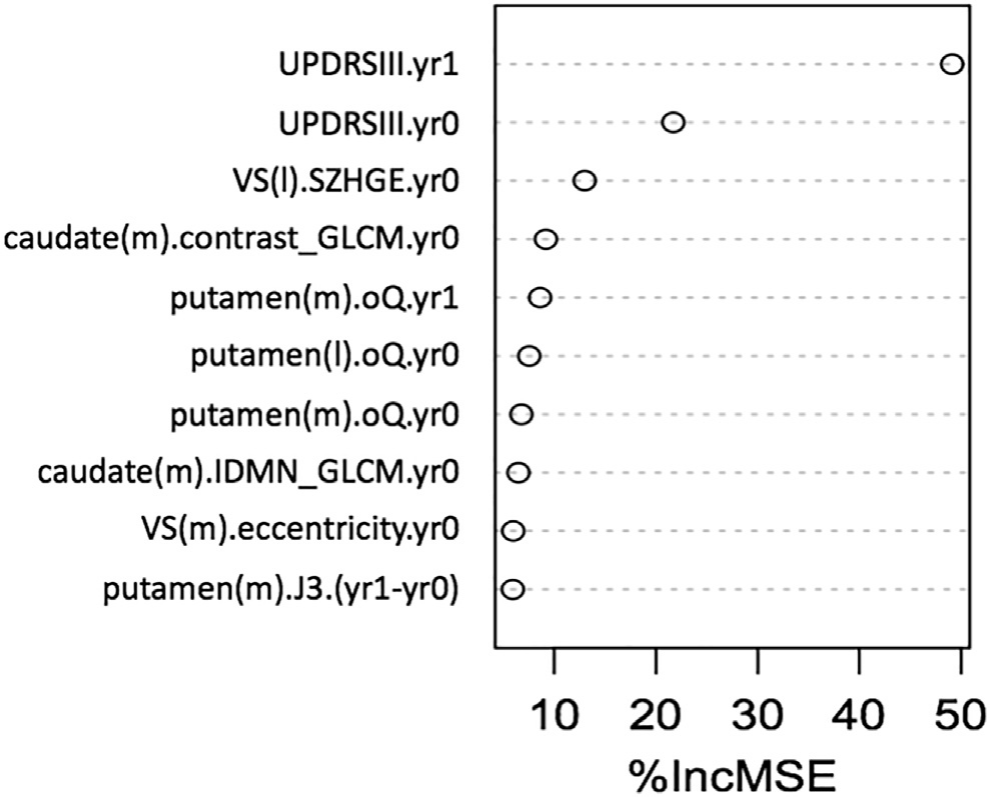
Relative contribution of different predictors. %IncMSE is incremental % change in mean square error (MSE) by exclusion of a single feature. Top two predictors are the clinical motor measures (UPDRS-III) in years 1 and 0. But as seen in fig. 3, additional use of the radiomic features is necessarily to significantly improve prediction of outcome. (m) and (l) refer to the more and less affected sides of a structure: putamen, caudate or VS (ventral striatum). The radiomic features are elaborated in the supplement.

Fig. 5 plots relative contribution of different predictors via a metric %IncMSE: incremental percent change in mean square error (MSE) by exclusion of a single feature. It is seen that UPDRS-III (motor) from year 1 and then from year 0 are the most important predictors. At the same time, it is seen that radiomics features were among the top 10 most important predictors. The critical observation, from Fig. 3, is that it is through the combination of conventional measures with radiomic features that one is able to significantly improve prediction of outcome.

## 4 Discussion

There exist two intertwined challenges in PD biomarker discovery. One is to identify biomarkers that track PD progression, and the other is to predict outcome (Marek et al., 2008). Both are very important clinically and in the discovery of disease modifying therapies. PD progression biomarkers are needed to assess efficacy of such therapies, while better prediction of outcome is needed in order to discover PD sub-types so as to more effectively design clinical trials in the first place.

In the present work, we utilized motor assessment (UPDRS-III) in year 4 as the outcome measure being predicted. We plan to extend our work to prediction of other outcomes, including cognitive assessment (MoCA) and time to initiation of symptomatic therapy (TIST). In fact, it is plausible that use of a single clinical metric may not be sufficient to truly track PD progression. As a result, it is meaningful to use a combination of metrics, or global statistical tests for assessment of disease progression in clinical trials (Huang et al., 2009), which we plan to utilize in future efforts involving prediction of outcome in PD.

There exist considerable difficulties and uncertainties with PD disease metrics. Disease duration is a particularly problematic metric, given the subtle and often nonspecific nature of early symptoms. The patients' abilities to detect the first symptoms is highly variable, and is impacted by a number of factors such as personality, education level and professional background, and the type of initial symptom (e.g. tremor vs. bradykinesia). This somewhat subjective nature of UPDRS evaluation makes the disease duration metric prone to inter-rater variability. UPDRS-III motor assessment itself is a highly variable measure (Horne et al., 2016, Mentzel et al., 2016), involving both patient- and rater-dependent variabilities. To reduce such variability, we averaged motor measures, if additionally performed up to 6 months before or after the visit. For instance, for year 4 outcome prediction, if a patient had a visit at 3.5 years and/or 4.5 years post-enrollment, the measures would be utilized and averaged with year 4 measures.

In our past efforts, we investigated use of advanced texture analysis in quantitative brain PET imaging, in studies of PD (Gonzalez et al., 2013, Klyuzhin et al., 2016, Sossi et al., 2012) and Neuroinflammation (Rahmim et al., 2012). Furthermore, we found strong evidence that such measures retain their information even as one transitions from the higher resolution domain of PET images to the lower resolution domain of SPECT images by significant post-reconstruction blurring of PET images (e.g. up to 1 cm) (Blinder et al., 2014). In a subsequent DAT SPECT study (Rahmim et al., 2016a), we also demonstrated significantly improved correlation of radiomic features with clinical assessment. This prompted the present study, for the challenging task of predicting clinical outcome, making use of radiomic features involving DAT SPECT images. It is also worth noting that a potential of radiomic features is to move beyond conventional measures that require access to a good reference region for normalization, enabling more subtle detection and assessment of disease (Campbell and Shokouhi, 2017).

The large number of features used in radiomics analysis is a concern in the context of the curse of dimensionality, and there exist increasing scrutiny (Chalkidou et al., 2015) and efforts (Kumar et al., 2012) to avoid problems of overfitting and false discovery. One approach is to pre-eliminate a number of features as shown in independent studies to depict poor repeatability (test-retest) or poor reproducibility to variations in image reconstruction and processing parameters (Ashrafinia et al., 2017, Doumou et al., 2015, Galavis et al., 2010, Grkovski et al., 2015, Hatt et al., 2013, Leijenaar et al., 2013, Lu et al., 2016, Lv et al., 2017, Shiri et al., 2017, Tixier et al., 2012, van Velden et al., 2016). An alternative approach, which we have pursued in this work, is to perform feature selection within the training dataset itself, using unsupervised methods (Bartenhagen et al., 2010, Dy, 2008, Mitra et al., 2002), to reduce the dimensionality of the data. This involved elimination of features that have very low dynamic range or those that are very highly correlated with other features (redundancy).

Among predictors used in this work, we also included genomics profiling. In particular, we used a reduced list of 8 alpha-synuclein (SNCA) single nucleotide polymorphisms (SNPs), given previous findings on their association with increased risk of PD onset (Guella et al., 2016). However, no significant genomic interactions were found for progression. Our ongoing efforts include correlation of the SNPs with imaging phenotypes to boost statistical power, and analysis of a wider array of SNPs. We plan on performing extensive analysis on a larger patient dataset, including a larger training set as well as separate validation set, in order to assess reproducibility of our findings of significantly improved prognosis with inclusion of radiomic features.

We finally note that there is increasing recognition for the critical role of standardization and reproducible research for effective progress in the hunt for and established utility of biomarkers (Collins and Tabak, 2014, Economist, 2013, Poste, 2011). This is equally a concern in the field of radiomics which involves complicated imaging feature definitions and analyses (Hatt et al., 2017). To this end, there are now significant efforts underway for standardization of imaging biomarkers (Zwanenburg et al., 2016), which are critically needed for reproducible application of radiomics features and their successful translation to clinical practice.

## 5 Conclusion

This work has shown that use of longitudinal data (year 0 and year 1), combined with radiomics analysis, can result in significant improvements in prediction of outcome. Our results demonstrated that addition of radiomic features to conventional measures significantly improved (*p* < 0.001) prediction of outcome (namely that of year 4 motor performance as assessed using MDS-UPDRS-III), reducing the absolute error of prediction from 9.00 ± 0.88 to 4.12 ± 0.43 (MDS-UPDRS-III distribution: 27.3 ± 14.7). Radiomics analysis of DAT SPECT images holds significant potential towards development of effective biomarkers for prognostication of PD, with implications in design of clinical trials.

## Conflicts of interest

None of the authors report any conflict of interest.

## Grant support

The project was supported by the Michael J. Fox Foundation, including use of data available from the PPMI—a public-private partnership—funded by The Michael J. Fox Foundation for Parkinson's Research and funding partners (listed at www.ppmi-info.org/fundingpartners). This work was also partially supported by the Natural Sciences and Engineering Research Council of Canada, and the National Natural Science Foundation of China (grant 61628105).

## Appendix A Definition of imaging features

Supplementary material. Image 1

## References

[bb0005] Aerts H.J.W.L., Velazquez E.R., Leijenaar R.T.H., Parmar C., Grossmann P., Carvalho S., Bussink J., Monshouwer R., Haibe-Kains B., Rietveld D., Hoebers F., Rietbergen M.M., Leemans C.R., Dekker A., Quackenbush J., Gillies R.J., Lambin P. (2014). Decoding tumour phenotype by noninvasive imaging using a quantitative radiomics approach. Nat. Commun..

[bb0010] Ashrafinia S., DiGianvittorio M., Rowe S., Gorin M., Lu L., Lodge M., Pomper M., Allaf M., Rahmim A. (2017). Reproducibility and reliability of radiomic features in ^18^F-DCFPyL PET/CT imaging of prostate cancer. J. Nucl. Med..

[bb0015] Asselin M.C., O'Connor J.P.B., Boellaard R., Thacker N.A., Jackson A. (2012). Quantifying heterogeneity in human tumours using MRI and PET. Eur. J. Cancer.

[bb0020] Badiavas K., Molyvda E., Iakovou I., Tsolaki M., Psarrakos K., Karatzas N. (2011). SPECT imaging evaluation in movement disorders: far beyond visual assessment. Eur. J. Nucl. Med. Mol. Imaging.

[bb0025] Bartenhagen C., Klein H.-U., Ruckert C., Jiang X., Dugas M. (2010). Comparative study of unsupervised dimension reduction techniques for the visualization of microarray gene expression data. BMC Bioinformatics.

[bb0030] Blinder S., Klyuzhin I., Gonzalez M.E., Rahmim A., Sossi V. (2014). Texture and shape analysis on high and low spatial resolution emission images. Proc. IEEE Nucl. Sci. Symp. Conf..

[bb0035] Brenneis C., Seppi K., Schocke M.F., Müller J., Luginger E., Bösch S., Löscher W.N., Büchel C., Poewe W., Wenning G.K. (2003). Voxel-based morphometry detects cortical atrophy in the Parkinson variant of multiple system atrophy. Mov. Disord..

[bb0040] Brooks D.J., Ibanez V., Sawle G.V., Quinn N., Lees A.J., Mathias C.J., Bannister R., Marsden C.D., Frackowiak R.S.J. (1990). Differing patterns of striatal F-18 Dopa uptake in Parkinsons-disease, multiple system atrophy, and progressive supranuclear palsy. Ann. Neurol..

[bb0045] Campbell D., Shokouhi S. (2017). Application of Haralick texture features in brain ^18^F-Florbetapir position emission tomography. J. Nucl. Med..

[bb0050] Catafau A.M., Tolosa E. (2004). Impact of dopamine transporter SPECT using I-123-ioflupane on diagnosis and management of patients with clinically uncertain Parkinsonian syndromes. Mov. Disord..

[bb0055] Chalkidou A., O'Doherty M.J., Marsden P.K. (2015). False discovery rates in PET and CT studies with texture features: a systematic review. PLoS One.

[bb0060] Chicklore S., Goh V., Siddique M., Roy A., Marsden P.K., Cook G.J.R. (2013). Quantifying tumour heterogeneity in F-18-FDG PET/CT imaging by texture analysis. Eur. J. Nucl. Med. Mol. Imaging.

[bb0065] Collignon A., Maes F., Delaere D., Vandermeulen D., Suetens P., Marchal G. (1995). Automated multi-modality image registration based on information theory. Information Processing in Medical Imaging.

[bb0070] Collins F.S., Tabak L.A. (2014). NIH plans to enhance reproducibility. Nature.

[bb0075] Djang D.S.W., Janssen M.J.R., Bohnen N., Booij J., Henderson T.A., Herholz K., Minoshima S., Rowe C.C., Sabri O., Seibyl J., Van Berckel B.N.M., Wanner M. (2012). SNM practice guideline for dopamine transporter imaging with I-123-Ioflupane SPECT 1.0. J. Nucl. Med..

[bb0080] Doumou G., Siddique M., Tsoumpas C., Goh V., Cook G.J. (2015). The precision of textural analysis in ^18^F-FDG-PET scans of oesophageal cancer. Eur. Radiol..

[bb0085] Dy J.G. (2008). Unsupervised Feature Selection. Computational Methods of Feature Selection.

[bb0090] Eary J.F., O'Sullivan F., O'Sullivan J., Conrad E.U. (2008). Spatial heterogeneity in sarcoma ^18^F-FDG uptake as a predictor of patient outcome. J. Nucl. Med..

[bb0095] Economist T. (2013). Unreliable research: trouble at the lab. Economist.

[bb0100] El Naqa I., Grigsby P.W., Apte A., Kidd E., Donnelly E., Khullar D., Chaudhari S., Yang D., Schmitt M., Laforest R., Thorstad W.L., Deasy J.O. (2009). Exploring feature-based approaches in PET images for predicting cancer treatment outcomes. Pattern Recogn..

[bb0105] Evans A.C., Collins D.L., Mills S., Brown E., Kelly R., Peters T.M. (1993). 3D statistical neuroanatomical models from 305 MRI volumes. Nuclear Science Symposium and Medical Imaging Conference, 1993.

[bb0110] Fischl B., Salat D.H., Busa E., Albert M., Dieterich M., Haselgrove C., Van Der Kouwe A., Killiany R., Kennedy D., Klaveness S. (2002). Whole brain segmentation: automated labeling of neuroanatomical structures in the human brain. Neuron.

[bb0115] Fischl B., Salat D.H., van der Kouwe A.J., Makris N., Ségonne F., Quinn B.T., Dale A.M. (2004). Sequence-independent segmentation of magnetic resonance images. NeuroImage.

[bb0120] Fischl B., Van Der Kouwe A., Destrieux C., Halgren E., Ségonne F., Salat D.H., Busa E., Seidman L.J., Goldstein J., Kennedy D. (2004). Automatically parcellating the human cerebral cortex. Cereb. Cortex.

[bb0125] Galavis P.E., Hollensen C., Jallow N., Paliwal B., Jeraj R. (2010). Variability of textural features in FDG PET images due to different acquisition modes and reconstruction parameters. Acta Oncol..

[bb0130] Garnett E.S., Lang A.E., Chirakal R., Firnau G., Nahmias C. (1987). A rostrocaudal gradient for aromatic acid decarboxylase in the human striatum. Can. J. Neurol. Sci..

[bb0135] Gonzalez M.E., Dinelle K., Vafai N., Heffernan N., McKenzie J., Appel-Cresswell S., McKeown M.J., Stoessl A.J., Sossi V. (2013). Novel spatial analysis method for PET images using 3D moment invariants: applications to Parkinson's disease. NeuroImage.

[bb0140] Grachev I., Kupsch A., Bajaj N., Weiland F., Tartaglione A., Klutmann S., Buitendyk M., Sherwin P., Tate A. (2012). Impact of DaTscan (TM) SPECT imaging on clinical management, diagnosis, and confidence of diagnosis in patients with clinically uncertain Parkinsonian syndromes: a prospective 1-year follow-up study. Neurology.

[bb0145] Grkovski M., Apte A., Schwartz J., Rimner A., Schoder H., Carlin S., Zanzonico P., Humm J., Nehmeh S. (2015). Reproducibility of F-18-FMISO intratumor distribution and texture features in NSCLC. J. Nucl. Med..

[bb0150] Guella I., Evans D.M., Szu-Tu C., Nosova E., Bortnick S.F., Goldman J.G., Dalrymple-Alford J.C., Geurtsen G.J., Litvan I., Ross O.A. (2016). α-Synuclein genetic variability: a biomarker for dementia in Parkinson disease. Ann. Neurol..

[bb0155] Hatt M., Tixier F., Le Rest C.C., Pradier O., Visvikis D. (2013). Robustness of intratumour ^18^F-FDG PET uptake heterogeneity quantification for therapy response prediction in oesophageal carcinoma. Eur. J. Nucl. Med. Mol. Imaging.

[bb0160] Hatt M., Majdoub M., Vallieres M., Tixier F., Le Rest C.C., Groheux D., Hindie E., Martineau A., Pradier O., Hustinx R., Perdrisot R., Guillevin R., El Naqa I., Visvikis D. (2015). F-18-FDG PET uptake characterization through texture analysis: investigating the complementary nature of heterogeneity and functional tumor volume in a multi-cancer site patient cohort. J. Nucl. Med..

[bb0165] Hatt M., Tixier F., Pierce L., Kinahan P.E., Le Rest C.C., Visvikis D. (2017). Characterization of PET/CT images using texture analysis: the past, the presenta… any future?. Eur. J. Nucl. Med. Mol. Imaging.

[bb0170] Horne M., Kotschet K., McGregor S. (2016). The clinical validation of objective measurement of movement in Parkinson's disease. CNS.

[bb0175] Huang P., Goetz C.G., Woolson R.F., Tilley B., Kerr D., Palesch Y., Elm J., Ravina B., Bergmann K.J., Kieburtz K. (2009). Using global statistical tests in long-term Parkinson's disease clinical trials. Mov. Disord..

[bb0180] Kish S.J., Shannak K., Hornykiewicz O. (1988). Uneven pattern of dopamine loss in the striatum of patients with idiopathic Parkinson's disease. Pathophysiologic and clinical implications. N. Engl. J. Med..

[bb0185] Klyuzhin I.S., Gonzalez M., Shahinfard E., Vafai N., Sossi V. (2016). Exploring the use of shape and texture descriptors of PET tracer distribution in imaging studies of neurodegenerative disease. J. Cereb. Blood Flow Metab..

[bb0190] Koch W., Radau P.E., Hamann C., Tatsch K. (2005). Clinical testing of an optimized software solution for an automated, observer-independent evaluation of dopamine transporter SPECT studies. J. Nucl. Med..

[bb0195] Kumar V., Gu Y.H., Basu S., Berglund A., Eschrich S.A., Schabath M.B., Forster K., Aerts H.J.W.L., Dekker A., Fenstermacher D., Goldgof D.B., Hall L.O., Lambin P., Balagurunathan Y., Gatenby R.A., Gillies R.J. (2012). Radiomics: the process and the challenges. Magn. Reson. Imaging.

[bb0200] Kupsch A.R., Bajaj N., Weiland F., Tartaglione A., Klutmann S., Buitendyk M., Sherwin P., Tate A., Grachev I.D. (2012). Impact of DaTscan SPECT imaging on clinical management, diagnosis, confidence of diagnosis, quality of life, health resource use and safety in patients with clinically uncertain parkinsonian syndromes: a prospective 1-year follow-up of an open-label controlled study. J. Neurol. Neurosurg. Psychiatry.

[bb0205] Lambin P., Rios-Velazquez E., Leijenaar R., Carvalho S., van Stiphout R.G.P.M., Granton P., Zegers C.M.L., Gillies R., Boellard R., Dekker A., Aerts H.J.W.L., Consortium Q.-C. (2012). Radiomics: extracting more information from medical images using advanced feature analysis. Eur. J. Cancer.

[bb0210] Leijenaar R.T.H., Carvalho S., Velazquez E.R., Van Elmpt W.J.C., Parmar C., Hoekstra O.S., Hoekstra C.J., Boellaard R., Dekker A.L.A.J., Gillies R.J., Aerts H.J.W.L., Lambin P. (2013). Stability of FDG-PET radiomics features: an integrated analysis of test-retest and inter-observer variability. Acta Oncol..

[bb0215] Lu L., Lv W., Jiang J., Ma J., Feng Q., Rahmim A., Chen W. (2016). Robustness of radiomic features in [^11^11C]choline and [^18^F]FDG PET/CT imaging of nasopharyngeal carcinoma: impact of segmentation and discretization. Mol. Imag. Biol..

[bb0220] Lv W., Lu L., Jiang J., Yang W., Ma J., Feng Q., Rahmim A., Chen W. (2017). Robustness of radiomic features in ^18^F-FDG PET/CT imaging of nasopharyngeal carcinoma: impact of parameter settings on different feature matrices. J. Nucl. Med..

[bb0225] Marek K., Jennings D., Tamagnan G., Seibyl J. (2008). Biomarkers for Parkison's disease: tools to assess Parkinson's disease onset and progression. Ann. Neurol..

[bb0230] Mentzel T.Q., Lieverse R., Levens A., Mentzel C.L., Tenback D.E., Bakker P.R., Daanen H.A., van Harten P.N. (2016). Reliability and validity of an instrument for the assessment of bradykinesia. Psychiatry Res..

[bb0235] Mitra P., Murthy C., Pal S.K. (2002). Unsupervised feature selection using feature similarity. IEEE Trans. Pattern Anal. Mach. Intell..

[bb0240] Parkinson Progression Marker Initiative (2011). The Parkinson progression marker initiative (PPMI). Prog. Neurobiol..

[bb0245] Parmar C., Grossmann P., Bussink J., Lambin P., Aerts H.J. (2015). Machine learning methods for quantitative radiomic biomarkers. Sci Rep.

[bb0250] Penny W.D., Friston K.J., Ashburner J.T., Kiebel S.J., Nichols T.E. (2011). Statistical Parametric Mapping: The Analysis of Functional Brain Images.

[bb0255] Poste G. (2011). Bring on the biomarkers. Nature.

[bb0260] Rahmim A., Coughlin J., Gonzalez M., Endres C.J., Zhou Y., Wong D.F., Wahl R.L., Sossi V., Pomper M.G. (2012). Novel parametric PET image quantification using texture and shape analysis.

[bb0265] Rahmim A., Salimpour Y., Jain S., Blinder S.A., Klyuzhin I.S., Smith G.S., Mari Z., Sossi V. (2016). Application of texture analysis to DAT SPECT imaging: relationship to clinical assessments. NeuroImage.

[bb0270] Rahmim A., Schmidtlein C.R., Jackson A., Sheikhbahaei S., Marcus C., Ashrafinia S., Soltani M., Subramaniam R.M. (2016). A novel metric for quantification of homogeneous and heterogeneous tumors in PET for enhanced clinical outcome prediction. Phys. Med. Biol..

[bb0275] Shiri I., Rahmim A., Ghaffarian P., Geramifar P., Abdollahi H., Bitarafan-Rajabi A. (2017). The impact of image reconstruction settings on ^18^F-FDG PET radiomic features: multi-scanner phantom and patient studies. Eur. Radiol..

[bb0280] Sossi V., Gonzalez M., Dinelle K., Heffernan N., McKenzie J., Cresswell S., McKeown M.J., Stoessl A.J. (2012). New insights into levodopa induced dopamine release in Parkinson's disease. J. Cereb. Blood Flow Metab..

[bb0285] Soufi M., Kamali-Asl A., Geramifar P., Rahmim A. (2017). A novel framework for automated segmentation and labeling of homogeneous versus heterogeneous lung tumors in [^18^F]FDG PET imaging. Mol. Imag. Biol..

[bb0290] Stoessl A.J., Martin W.R.W., McKeown M., Sossi V. (2011). Advances in imaging in Parkinson's disease. Lancet Neurol..

[bb0295] Tixier F., Le Rest C.C., Hatt M., Albarghach N., Pradier O., Metges J.P., Corcos L., Visvikis D. (2011). Intratumor heterogeneity characterized by textural features on baseline ^18^F-FDG PET images predicts response to concomitant Radiochemotherapy in esophageal cancer. J. Nucl. Med..

[bb0300] Tixier F., Hatt M., Le Rest C.C., Le Pogam A., Corcos L., Visvikis D. (2012). Reproducibility of tumor uptake heterogeneity characterization through textural feature analysis in F-18-FDG PET. J. Nucl. Med..

[bb0305] Tixier F., Hatt M., Valla C., Fleury V., Lamour C., Ezzouhri S., Ingrand P., Perdrisot R., Visvikis D., Le Rest C.C. (2014). Visual versus quantitative assessment of intratumor F-18-FDG PET uptake heterogeneity: prognostic value in non-small cell lung cancer. J. Nucl. Med..

[bb0310] van Velden F.H.P., Cheebsumon P., Yaqub M., Smit E.F., Hoekstra O.S., Lammertsma A.A., Boellaard R. (2011). Evaluation of a cumulative SUV-volume histogram method for parameterizing heterogeneous intratumoural FDG uptake in non-small cell lung cancer PET studies. Eur. J. Nucl. Med. Mol. Imaging.

[bb0315] van Velden F.H., Kramer G.M., Frings V., Nissen I.A., Mulder E.R., de Langen A.J., Hoekstra O.S., Smit E.F., Boellaard R. (2016). Repeatability of radiomic features in non-small-cell lung cancer [^18^F] FDG-PET/CT studies: impact of reconstruction and delineation. Mol. Imaging Biol..

[bb0320] Vriens D., Disselhorst J.A., Oyen W.J.G., de Geus-Oei L.F., Visser E.P. (2012). Quantitative assessment of heterogeneity in tumor metabolism using FDG-PET. Int. J. Radiat. Oncol. Biol. Phys..

[bb0325] Zeighami Y., Ulla M., Iturria-Medina Y., Dadar M., Zhang Y., Larcher K.M.-H., Fonov V., Evans A.C., Collins D.L., Dagher A. (2015). Network structure of brain atrophy in de novo Parkinson's disease. elife.

[bb0330] Zwanenburg A., Leger S., Vallières M., Löck S. (2016). Image biomarker standardisation initiative — feature definitions. CoRR.

