# Supplementary material for: Improved prediction of outcome in Parkinson's disease using radiomics analysis of longitudinal DAT SPECT images
Source: Neuroimage Clin. 2017 Aug 26;16:539–44. doi: 10.1016/j.nicl.2017.08.021 (PMC5984570; doi:10.1016/j.nicl.2017.08.021)
Supplement: Supplementary file 1 — Supplementary material. [file mmc1.docx]

**Supplementary Material**

Appendix A. Definition of imaging features

For a given region of interest (ROI), various features are extracted, as follows.

- **First order gray level statistics features - 13**

Let $P$ define the first-order histogram of tumor volume. $P(i)$ represents the number of voxels with gray level $i$, and $N_{g}$ represents the number of gray-level bins set for $P$. The $i^{th}$ entry of the normalized histogram is then defined as:

$$p(i)=\frac{P(i)}{\sum_{i=1}^{N_{g}} P(i)}$$

1. SUVmax: the maximum SUV value.
2. SUVmean: the mean SUV value.
3. SUVpeak: defined as the mean SUV within a 26 connected neighborhoods volume centered the maximum SUV voxel.
4. SUVstd: the standard deviation of all SUV values.
5. SUVvar: the variance of all SUV values.
6. SUVenergy: the sum of all voxel SUV values squared.
7. AUC-CSH: Area under the curve of the cumulative SUV-volume histogram describing the percentage of total tumor volume above a percentage threshold of maximum SUV (1)
8. Mean_hist:

$$\mu=\sum_{i=1}^{Ng} ip(i)$$

1. Variance_hist:

$$\sigma^{2}=\sum_{i=1}^{N_{g}} (i-\mu)^{2}p(i)$$

1. Skewness_hist:

$$s=\sigma^{-3}\sum_{i=1}^{N_{g}} (i-\mu)^{3}p(i)$$

1. Kurtosis_hist:

$$k=\sigma^{-4}\sum_{i=1}^{N_{g}} (i-\mu)^{4}p(i)-3$$

1. Energy_hist:

$$energy\_hist=\sum_{i=1}^{N_{g}} p(i)^{2}$$

1. Entropy_hist:

$$entropy\_hist=-\sum_{i=1}^{Ng} p(i)\log_{2}[p(i)]$$

- **Morphological features - 22**

Morphological features, describing the shape and size of the volume of interest. Of these 22 features, 17 of them (all but features 26-30) are purely based on the anatomical structure, while features 26-30 also take into account the uptake intensity within the ROI.

Let $V$ be the volume and $A$ the surface area of the volume of interest.

1. ROI-Volume: volume of the ROI
2. TLG: total lesion glycolysis, defined as the product of Volume and SUVmean.
3. GT2: Same as TLG, except only voxels with intensity at least 2 times the reference region are included (11).
4. Eccentricity: find an ellipsoid that best fits the tumor region, and the eccentricity is then given by $(1-a\times\frac{b}{c^{2}})^{\frac{1}{2}}$, where $c$ is the longest semi-principal axes of the ellipsoid, $a$ and $b$ are the second and third longest semi-principal axes of the ellipsoid.
5. Solidity: ratio of the number of voxels in the tumor region to the number of voxels in the 3D convex hull of the tumor region (smallest polyhedron containing the tumor region).
6. PI: percent inactive, percentage of the tumor region that is inactive. A threshold of $0.005\times SUV\max^{2}$ followed by closing and opening morphological operations were used to differentiate active and inactive regions on PET scans.
7. Surface: the surface area of the volume of interest.
8. SVratio: the surface area divided by the volume.
9. Compactness 1:

$$compactness 1=\frac{\text{V}}{\sqrt{\pi}\text{A}^{\frac{2}{3}}}$$

1. Compactness 2:

$$compactness 2=36\pi\frac{V^{2}}{\text{A}^{3}}$$

1. Sphericity：

$$sphericity=\frac{\pi^{\frac{1}{3}}\left(6V\right)^{\frac{2}{3}}}{\text{A}}$$

1. Size-ROI: Maximum 3D diameter of the ROI.

We also include 3D moment invariants (3D-MIs) (9). MIs are mathematical spatial descriptors designed to be invariant to scaling, translation and rotation, with extensive use in a number of areas including pattern. Let us define:

$$\mu_{pqr}=\int_{-\infty}^{\infty} \int_{-\infty}^{\infty} \int_{-\infty}^{\infty} \left( x-\bar{x} \right)^{p}\left( y-\bar{y} \right)^{q}\left( z-\bar{z} \right)^{r}f\left( x,y,z \right)dxdydz$$

$$\eta_{pqr}=\frac{\mu_{pqr}}{\mu_{000}^{\frac{p+q+r}{3}+1}}$$

where f(x,y,z) refers to the uptake intensity within the ROI, with all values outside of ROI set to 0, thus integration only takes places within the ROI. Then we generate second-order MIs (9,10):

$J_{1}=\eta_{200}+\eta_{020}+\eta_{002}$

$J_{2}=\eta_{200}\eta_{020}+\eta_{200}\eta_{002}+\eta_{020}\eta_{002}-\eta_{101}^{2}-\eta_{110}^{2}-\eta_{011}^{2}$

$J_{3}=\eta_{200}\eta_{020}\eta_{002}-\eta_{002}\eta_{110}^{2}-\eta_{020}\eta_{101}^{2}-\eta_{200}\eta_{011}^{2}+2\eta_{110}\eta_{101}\eta_{011}$

1. Q which turns out to be related to J1 and J2 according to: $J_{2}=\frac{1}{2}\left( J_{1}^{2}-Q \right)$

We also produce a third-order moment invariant:

$B_{3}=\eta_{300}^{2}+\eta_{030}^{2}+\eta_{003}^{2}+3\left( \eta_{210}^{2}+\eta_{021}^{2}+\eta_{201}^{2}+\eta_{120}^{2}+\eta_{012}^{2}+\eta_{102}^{2} \right)+6\eta_{111}^{2}$

We also generate an intensity-independent definition, where f(x,y,z) is set uniformly to 1 in above definition, and 0 otherwise, to generate metrics analogous to above definitions though independent of uptake, and only dependent on the ROI shape:

${oJ}_{1}$

$oJ_{2}$

${oJ}_{3}$

1. oQ

${oB}_{3}$

- **Second- and higher-order textural features – 57**

This class of features consists of four sub-groups:

**1) Gray Level Co-occurrence Matrix-based features (GLCM) - 26**

Gray level co-occurrence matrix-based features, as described by study (2). Let: $P(i,j)$ be the co-occurrence matrix, $N_{g}$ be the number of discrete intensity levels in the image, $\mu$ be the mean of $P(i,j)$, $\mu_{x}(i)$ be the mean of row $i$, $\mu_{x}(j)$be the mean of column $j$, $\sigma_{x}(i)$ be the standard deviation of row $i$, $\sigma_{y}(j)$ be the standard deviation of column $j$.

$$p_{x}\left(i\right)=\sum_{j=1}^{N_{g}} P\left(i,j\right)$$

$$p_{y}\left(j\right)=\sum_{i=1}^{N_{g}} P\left(i,j\right)$$

$$p_{x+y}\left(k\right)=\sum_{i=1}^{N_{g}} \sum_{j=1}^{N_{g}} P\left(i,j\right), i+j=k,k=2,3,\ldots2N_{g}$$

$$p_{x-y}\left(k\right)=\sum_{i=1}^{N_{g}} \sum_{j=1}^{N_{g}} P\left(i,j\right), i-j=k,k=0,1,\ldots N_{g}-1$$

$$HXY1=-\sum_{i=1}^{N_{g}} \sum_{j=1}^{N_{g}} P\left(i,j\right)\log\left(p_{x}\left(i\right)p_{y}\left(j\right)\right)$$

$$HXY2=-\sum_{i=1}^{N_{g}} \sum_{j=1}^{N_{g}} P_{x}\left(i\right)P_{y}\left(j\right)\log\left(p_{x}\left(i\right)p_{y}\left(j\right)\right)$$

$$HX=-\sum_{i=1}^{N_{g}} p_{x}(i)\log_{2}[p_{x}(i)]$$

$$HY=-\sum_{i=1}^{N_{g}} p_{y}(i)\log_{2}[p_{y}(i)]$$

$$H=-\sum_{i=1}^{N_{g}} \sum_{j=1}^{N_{g}} p(i,j)\log_{2}[p(i,j)]$$

1. Energy, called Uniformity in (3), also called Angular second moment in (4):

$$energy=\sum_{i=1}^{N_{g}} \sum_{j=1}^{N_{g}} \left[P\left(i,j\right)\right]^{2}$$

1. Entropy:

$$entropy=-\sum_{i=1}^{N_{g}} \sum_{j=1}^{N_{g}} P\left(i,j\right)\log_{2}\left[P\left(i,j\right)\right]$$

1. Difference entropy (DifEntropy):

$$difference entropy=-\sum_{i=0}^{N_{g}-1} P_{x-y}\left(i\right)\log_{2}\left[P_{x-y}\left(i\right)\right]$$

1. Sum entropy (SumEntropy):

$$sum entropy=-\sum_{i=2}^{2N_{g}} P_{x+y}\left(i\right)\log_{2}\left[P_{x+y}\left(i\right)\right]$$

1. Variance1:

$$variance1=\frac{1}{N_{g}\times N_{g}}\sum_{i=1}^{N_{g}} \sum_{j=1}^{N_{g}} [(i-\mu_{x})^{2}p(i,j)+(j-\mu_{y})^{2}p(i,j)]$$

1. Variance2:

$$variance2=\sum_{i=1}^{N_{g}} \sum_{j=1}^{N_{g}} \left(i-\mu\right)^{2}P\left(i,j\right)$$

1. Sum variance (SumVariance):

$$sum variance=\sum_{i=2}^{2N_{g}} \left(i-SE\right)^{2}P_{x+y}\left(i\right)$$

Where SE is Sum entropy

1. Maximum probability (MaxPossilility):

$$maximum probability=\max\left\{P\left(i,j\right)\right\}$$

1. Contrast:

$$contrast=\sum_{i=1}^{N_{g}} \sum_{j=1}^{N_{g}} \left|i-j\right|^{2}P\left(i,j\right)$$

1. Dissimilarity:

$$dissimilarity=\sum_{i=1}^{N_{g}} \sum_{j=1}^{N_{g}} \left|i-j\right|P\left(i,j\right)$$

1. Homogeneity 1, also called Inverse difference in (3):

$$homogeneity 1=\sum_{i=1}^{N_{g}} \sum_{j=1}^{N_{g}} \frac{P\left(i,j\right)}{1+\left|i-j\right|}$$

1. Homogeneity 2, also called local homogeneity in (5):

$$homogeneity 2=\sum_{i=1}^{N_{g}} \sum_{j=1}^{N_{g}} \frac{P\left(i,j\right)}{1+\left|i-j\right|^{2}}$$

1. Correlation1:

$$correlation1=\sum_{i=1}^{N_{g}} \sum_{j=1}^{N_{g}} \frac{(i-\mu_{x})(j-\mu_{y})p(i,j)}{\sigma_{x}\sigma_{y}}$$

1. Correlation2:

$$correlation2=\frac{\sum_{i=1}^{N_{g}} \sum_{j=1}^{N_{g}} ijP\left(i,j\right)-\mu_{x}\left(i\right)\mu_{y}\left(j\right)}{\sigma_{x}\left(i\right)\sigma_{y}\left(j\right)}$$

1. Auto correlation (AutoCorrelation):

$$auto correlation=\sum_{i=1}^{N_{g}} \sum_{j=1}^{N_{g}} ijp\left(i,j\right)$$

1. Cluster prominence (ClusterPro):

$$cluster prominence=\sum_{i=1}^{N_{g}} \sum_{j=1}^{N_{g}} \left[i+j-\mu_{x}\left(i\right)-\mu_{y}\left(j\right)\right]^{4}P\left(i,j\right)$$

1. Cluster shade (ClusterShade):

$$cluster shade=\sum_{i=1}^{N_{g}} \sum_{j=1}^{N_{g}} \left[i+j-\mu_{x}\left(i\right)-\mu_{y}\left(j\right)\right]^{3}P\left(i,j\right)$$

1. Cluster tendency (ClusterTen):

$$cluster tendency=\sum_{i=1}^{N_{g}} \sum_{j=1}^{N_{g}} \left[i+j-\mu_{x}\left(i\right)-\mu_{y}\left(j\right)\right]^{2}P\left(i,j\right)$$

1. Informational measure of correlation 1 (IMC1):

$$IMC1=\frac{H-HXY1}{\max\left\{HX,HY\right\}}$$

Where *HX and HY* are the entropies of $p_{x}$ and $p_{y}$.

1. Informational measure of correlation 2 (IMC2):

$$IMC2=\sqrt{1-e^{-2\left(HXY2-H\right)}}$$

where *H* is the entropy.

1. Inverse difference moment (InvDifMoment) also called inverse variance:

$$inverse difference moment=\sum_{i=1}^{N_{g}} \sum_{j=1}^{N_{g}} \frac{P\left(i,j\right)}{\left|i-j\right|^{2}},i\neq j$$

1. Inverse Difference Moment Normalized (IDMN):

$$IDMN=\sum_{i=1}^{N_{g}} \sum_{j=1}^{N_{g}} \frac{P\left(i,j\right)}{1+\left(\frac{\left|i-j\right|^{2}}{N^{2}}\right)}$$

1. Inverse Difference Normalized (IDN):

$$IDN=\sum_{i=1}^{N_{g}} \sum_{j=1}^{N_{g}} \frac{P\left(i,j\right)}{1+\left(\frac{\left|i-j\right|}{N}\right)}$$

1. Sum average1:

$$sum average1=\frac{1}{N_{g}\times N_{g}}\sum_{i=1}^{N_{g}} \sum_{j=1}^{N_{g}} [iP(i,j)+jP(i,j)]$$

1. Sum average2:

$$sum average2=\sum_{i=2}^{2N_{g}} \left[iP_{x+y}\left(i\right)\right]$$

1. Agreement:

$$agreement=\frac{P_{o}-P_{e}}{1-P_{e}}$$

where $P_{o}=\sum_{i=1}^{Ng} P(i,i)$ $P_{e}=\sum_{i=1}^{Ng} P(i,:)P(:,i)$

**2) Gray Level Run Length Matrix-based features (GLRLM)-13**

Gray-level run-length matrix-based features, as described by Galloway et al.(6). Let: $P(i,j)$ be the $(i,j)th$ entry in the given run-length matrix, $N_{g}$ the number of discrete intensity values in the image, $N_{r}$the number of different run lengths, $N_{p}$ is the number of voxels in the image, and the entry $(i,j)$ of the normalized GLRLM is defined as:

$$p(i,j)=\frac{P(i,j)}{\sum_{i=1}^{N_{g}} \sum_{j=1}^{N_{r}} P(i,j)}$$

$$\mu_{i}=\sum_{i=1}^{N_{g}} i\sum_{j=1}^{N_{r}} p(i,j)$$

$$\mu_{j}=\sum_{j=1}^{N_{r}} j\sum_{i=1}^{N_{g}} p(i,j)$$

1. Short Run Emphasis (SRE):

$$SRE=\sum_{i=1}^{N_{g}} \sum_{j=1}^{N_{r}} \left[\frac{p\left(i,j\right)}{j^{2}}\right]$$

1. Long Run Emphasis (LRE):

$$LRE=\sum_{i=1}^{N_{g}} \sum_{j=1}^{N_{r}} j^{2}p\left(i,j\right)$$

1. Gray Leven Non-Uniformity (GLN):

$$GLN=\sum_{i=1}^{N_{g}} \left[\sum_{j=1}^{N_{r}} p\left(i,j\right)\right]^{2}$$

1. Run Length Non-Uniformity (RLN):

$$RLN=\sum_{i=1}^{N_{r}} \left[\sum_{j=1}^{N_{g}} p\left(i,j\right)\right]^{2}$$

1. Run Percentage (RP):

$$RP=\sum_{i=1}^{N_{g}} \sum_{j=1}^{N_{r}} \frac{p\left(i,j\right)}{N_{p}}$$

1. Low Gray Level Run Emphasis (LGRE):

$$LGRE=\sum_{i=1}^{N_{g}} \sum_{j=1}^{N_{r}} \left[\frac{p\left(i,j\right)}{i^{2}}\right]$$

1. High Gray Level Run Emphasis (HGRE):

$$HGRE=\sum_{i=1}^{N_{g}} \sum_{j=1}^{N_{r}} i^{2}p\left(i,j\right)$$

1. Short Run Low Gray Level Emphasis (SRLGE):

$$SRLGE=\sum_{i=1}^{N_{g}} \sum_{j=1}^{N_{r}} \left[\frac{p\left(i,j\right)}{i^{2}j^{2}}\right]$$

1. Short Run High Gray Level Emphasis (SRHGE):

$$SRHGE=\sum_{i=1}^{N_{g}} \sum_{j=1}^{N_{r}} \left[\frac{p\left(i,j\right)i^{2}}{j^{2}}\right]$$

1. Long Run Low Gray Level Emphasis (LRLGE):

$$LRLGE=\sum_{i=1}^{N_{g}} \sum_{j=1}^{N_{r}} \left[\frac{p\left(i,j\right)j^{2}}{i^{2}}\right]$$

1. Long Run High Gray Level Emphasis (LRHGE):

$$LRHGE=\sum_{i=1}^{N_{g}} \sum_{j=1}^{N_{r}} p\left(i,j\right)i^{2}j^{2}$$

1. Gray Level Variance (GLV)

$$GLV=\frac{1}{N_{g}\times N_{r}}\sum_{i=1}^{N_{g}} \sum_{j=1}^{N_{r}} (ip(i,j)-\mu_{i})^{2}$$

1. Run length Variance (RLV)

$$RLV=\frac{1}{N_{g}\times N_{r}}\sum_{i=1}^{N_{g}} \sum_{j=1}^{N_{r}} (jp(i,j)-\mu_{j})^{2}$$

**3) Gray Level Size Zone Matrix-based features (GLSZM)-13**

Gray-level size-zone matrix-based features, was described in (2). Let: $P(i,j)$ be the $(i,j)th$ entry in the given size-zone matrix, $N_{g}$ the number of discrete intensity values in the image, $N_{z}$ the size of the largest homogeneous region in the volume of interest, $N_{\alpha}$ the number homogeneous zones in the image. The entry $(i,j)$ of the GLSZM then normalized as:

$$p(i,j)=\frac{P(i,j)}{\sum_{i=1}^{N_{g}} \sum_{j=1}^{N_{z}} P(i,j)}$$

$$\mu_{i}=\sum_{i=1}^{N_{g}} i\sum_{j=1}^{N_{z}} p(i,j)$$

$$\mu_{j}=\sum_{j=1}^{N_{z}} j\sum_{i=1}^{N_{g}} p(i,j)$$

1. Small Zone Emphasis (SZE):

$$SZE=\sum_{i=1}^{N_{g}} \sum_{j=1}^{N_{z}} \left[\frac{p\left(i,j\right)}{j^{2}}\right]$$

1. Large Zone Emphasis (LZE):

$$LZE=\sum_{i=1}^{N_{g}} \sum_{j=1}^{N_{z}} j^{2}p\left(i,j\right)$$

1. Gray Level Non-uniformity (GLN) also called Intensity Variability (IV) in (7):

$$GLN=\sum_{i=1}^{N_{g}} \left[\sum_{j=1}^{N_{z}} p\left(i,j\right)\right]^{2}$$

1. Zone Size Non-uniformity (ZSN) also called Size Zone Variability (SZV) in (7):

$$ZSN=\sum_{i=1}^{N_{z}} \left[\sum_{j=1}^{N_{g}} p\left(i,j\right)\right]^{2}$$

1. Zone Percentage (ZP):

$$ZP=\sum_{i=1}^{N_{z}} \sum_{j=1}^{N_{g}} \frac{p\left(i,j\right)}{N_{\alpha}}$$

1. Low Gray Level Zone Emphasis (LGZE) also called Low Intensity Emphasis (LIE) in (7):

$$LGZE=\sum_{i=1}^{N_{g}} \sum_{j=1}^{N_{z}} \left[\frac{p\left(i,j\right)}{i^{2}}\right]$$

1. High Gray level Zone Emphasis (HGZE) also called High Intensity Emphasis (HIE) in (7):

$$HGZE=\sum_{i=1}^{N_{g}} \sum_{j=1}^{N_{z}} i^{2}p\left(i,j\right)$$

1. Small Zone Low Gray Level Emphasis (SZLGE) also called Low Intensity Small Area Emphasis (LISAE) in (7):

$$SZLGE=\sum_{i=1}^{N_{g}} \sum_{j=1}^{N_{z}} \left[\frac{p\left(i,j\right)}{i^{2}j^{2}}\right]$$

1. Small Zone High Gray-Level Emphasis (SZHGE) also called High Intensity Small Area Emphasis (HISAE) in (7):

$$SZHGE=\sum_{i=1}^{N_{g}} \sum_{j=1}^{N_{z}} \left[\frac{p\left(i,j\right)i^{2}}{j^{2}}\right]$$

1. Large Zone Low Gray-Level Emphasis (LZLGE) also called Low Intensity Large Area Emphasis (LILAE) in (7):

$$LZLGE=\sum_{i=1}^{N_{g}} \sum_{j=1}^{N_{z}} \left[\frac{p\left(i,j\right)j^{2}}{i^{2}}\right]$$

1. Large Zone High Gray-Level Emphasis (LZHGE) also called High Intensity Large Area Emphasis (HILAE) in (7):

$$LZHGE=\sum_{i=1}^{N_{g}} \sum_{j=1}^{N_{z}} p\left(i,j\right)i^{2}j^{2}$$

1. Gray Level Variance (GLV)

$$GLV=\frac{1}{N_{g}\times N_{z}}\sum_{i=1}^{N_{g}} \sum_{j=1}^{N_{z}} (ip(i,j)-\mu_{i})^{2}$$

1. Zone Size Variance (ZSV)

$$ZSV=\frac{1}{N_{g}\times N_{z}}\sum_{i=1}^{N_{g}} \sum_{j=1}^{N_{z}} (jp(i,j)-\mu_{j})^{2}$$

where zone aforesaid also called area in (7).

**4) Neighborhood Gray Tone Difference Matrix–based features (NGTDM) - 5**

NGTDM is a column matrix (8), Let$i^{th}$entry of the NGTDM is$P(i)$, defined as:

$$P(i)=\left\{\begin{matrix} \sum_{i\in\left\{\left.N_{i}\right\}\right.} \left|i-\overline{A_{i}}\right|\text{ }if\text{ }N_{i}\text{>0,} \\ 0\text{ }otherwise. \end{matrix}\right.$$

where $\{N_{i}\}$ is the set of all voxels with gray-level $i$ in tumor volume (including the peripheral region), $N_{i}$is the number of voxels with gray-level $i$ in tumor volume, and $A_{i}$ is the average gray level of the 26-connected neighbors around a center voxel $V(i,j,k)$ with gray level $i$.

$$\overline{A_{i}}=\overline{A(}j,k,l)=\frac{1}{W}\sum_{m=-d}^{d} \sum_{n=-d}^{d} \sum_{s=-d}^{d} V(j+m,k+n,l+s),(m,n,l)\neq(0,0,0)$$

where $d=1$, specifies the neighborhood size as $3\times3\times3$, and $W=(2d+1)^{3}$, The quantity $n_{i}=\frac{N_{i}}{N}$ is also defined, where $N$ is the total number of voxels in tumor volume. The NGTDM texture features are then defined as:

1. Coarseness:

$$coarseness=[\varepsilon+\sum_{i=1}^{N_{g}} n_{i}P(i)]^{-1}$$

where $\varepsilon$ is a small number to prevent coarseness becoming infinite, *N_g_* the number of discrete intensity values in the image.

1. Contrast:

$$contrast=[\frac{1}{N_{g}\times(N_{g}-1)}\sum_{i=1}^{N_{g}} \sum_{j=1}^{N_{g}} n_{i}n_{j}(i-j)^{2}]\times[\frac{1}{N}\sum_{i=1}^{N_{g}} P(i)]$$

1. Busyness:

$$busyness=\frac{\sum_{i=1}^{N_{g}} n_{i}P(i)}{\sum_{i=1}^{N_{g}} \sum_{j=1}^{N_{g}} \left|in_{i}-jn_{j}\right|},n_{i}\neq0,n_{j}\neq0$$

1. Complexity:

$$complexity=\sum_{i=1}^{N_{g}} \sum_{j=1}^{N_{g}} \frac{\left|i-j\right|\left[n_{i}P(i)+n_{j}P(j)\right]}{N(n_{i}+n_{j})},n_{i}\neq0,n_{j}\neq0$$

1. Strength:

$$strength=\frac{\sum_{i=1}^{N_{g}} \sum_{j=1}^{N_{g}} (n_{i}-n_{j})(i-j)^{2}}{\varepsilon+\sum_{i=1}^{N_{g}} P(i)},n_{i}\neq0,n_{j}\neq0$$

where $\varepsilon$ is a small number to prevent strength becoming infinite.

**References:**

1. van Velden, F. H., *et al.* 2011. Evaluation of a cumulative SUV-volume histogram method for parameterizing heterogeneous intratumoural FDG uptake in non-small cell lung cancer PET studies. *Eur J Nucl Med Mol Imaging* 38 (9):1636-47.

2. Thibault, G., *et al*. 2009. Texture indexes and gray level size zone matrix application to cell nuclei classification.

3. Gomez, W., W. C. Pereira, and A. F. Infantosi. 2012. Analysis of co-occurrence texture statistics as a function of gray-level quantization for classifying breast ultrasound. *IEEE Trans Med Imaging* 31 (10):1889-99.

4. Lee, J., *et al*. 2015. Texture Feature Ratios from Relative CBV Maps of Perfusion MRI Are Associated with Patient Survival in Glioblastoma. *AJNR Am J Neuroradiol*.

5. El Naqa I., *et al*. 2009. Exploring feature-based approaches in PET images for predicting cancer treatment outcomes. *Pattern Recognit* 42 (6):1162-1171.

6. Galloway, M. M. 1974. Texture analysis using grey level run lengths. *NASA STI/Recon Technical Report N* 75:18555.

7. Leijenaar, R. T. H., *et al.* 2013. Stability of FDG-PET Radiomics features: an integrated analysis of test-retest and inter-observer variability. *Acta Oncol* 52 (7):1391-7.

8. Amadasun, M., and King, R. 1989. Textural features corresponding to textural properties. *Systems, Man and Cybernetics, IEEE Transactions on* 19 (5):1264-1274.

9. Flusser, J., Suk, T. and Zitova, B. Moments and moment invariants in pattern recognition. (*Wiley & Sons, Inc.,* 2009).

10. Gonzalez, M. E., *et al.* Novel spatial analysis method for PET images using 3D moment invariants: Applications to Parkinson's disease. 2013. *Neuroimage* 68:11-21.

11. Shenkov, N., *et al.* A metric to quantify DaTSCAN tracer uptake in subjects with Parkinson’s disease computed without MRI-based regions of interest. 2017. *J Nucl Med* 58 (suppl. 1):291.
